# Salicylic acid interferes with GFP fluorescence *in vivo*

**DOI:** 10.1093/jxb/erx031

**Published:** 2017-03-29

**Authors:** Jennifer de Jonge, Daniel Hofius, Lars Hennig

**Affiliations:** 1 Swedish University of Agricultural Sciences, Department of Plant Biology and Linnean Center for Plant Biology, PO Box 7080, SE-75007 Uppsala, Sweden

**Keywords:** Arabidopsis, fluorescence microscopy, fluorescent protein, GFP fusion proteins, histones, salicylic acid.

## Abstract

Fluorescent proteins have become essential tools for cell biologists. They are routinely used by plant biologists for protein and promoter fusions to infer protein localization, tissue‐specific expression and protein abundance. When studying the effects of biotic stress on chromatin, we unexpectedly observed a decrease in GFP signal intensity upon salicylic acid (SA) treatment in Arabidopsis lines expressing histone H1-GFP fusions. This GFP signal decrease was dependent on SA concentration. The effect was not specific to the linker histone H1-GFP fusion but was also observed for the nucleosomal histone H2A-GFP fusion. This result prompted us to investigate a collection of fusion proteins, which included different promoters, subcellular localizations and fluorophores. In all cases, fluorescence signals declined strongly or disappeared after SA application. No changes were detected in GFP‐fusion protein abundance when fluorescence signals were lost indicating that SA does not interfere with protein stability but GFP fluorescence. *In vitro* experiments showed that SA caused GFP fluorescence reduction only *in vivo* but not *in vitro*, suggesting that SA requires cellular components to cause fluorescence reduction. Together, we conclude that SA can interfere with the fluorescence of various GFP‐derived reporter constructs *in vivo*. Assays that measure relocation or turnover of GFP‐tagged proteins upon SA treatment should therefore be evaluated with caution.

## Highlight

Salicylic acid (SA) interferes with fluorescence from GFP‐derived reporter constructs *in planta* and as such assays using these constructs with SA treatment should be evaluated cautiously.

## Introduction

The green fluorescent protein (GFP) discovered in the jellyfish *Aequorea victoria* became universally famous in science after it was cloned (Prasher *et al.*, 1992) and used as an *in vivo* fluorescent tag (Chalfie *et al.*, 1994) for proteins in animals, yeast cells and higher plants (Cubitt *et al.*, 1995). Soon after, red fluorescent proteins (RFPs) were cloned and developed into fluorescent probes (Matz *et al.*, 1999). Around the same time mutation studies led to the availability of different GFP variants with improved characteristics (Tsien, 1998). Since then it has become common practice to fuse proteins of interest to fluorescent tags in order to analyze protein location (Tsien, 1998), expression (van Roessel and Brand, 2002), and host pathogen-interactions, as well as many other applications. Understanding transcriptional timing and protein expression is of utmost importance when characterizing the induction of reporter genes or analyzing unknown promoter sequences (Chalfie *et al.*, 1994). In *Arabidopsis thaliana*, gene expression and/or promotor activation studies utilizing GFP fusion proteins are well established and are even possible in a high-throughput manner (Xiao *et al.*, 2010).

Plants need to constantly adapt their growth and metabolism to varying environmental cues. Studying the regulatory pathways of gene activation helps unravel plant responses to abiotic and biotic stresses. Several pathways of plant responses to stress have been discovered during the last few decades, including chromatin-based mechanisms that contribute to abiotic stress responses (Hennig, 2012; Sani *et al.*, 2013; Shen *et al.*, 2014; Mehdi *et al.*, 2015; Vriet *et al.*, 2015).

Linker histones play important roles in higher-order chromatin compaction in eukaryotes and are the most variable of the histone family proteins (Syed *et al.*, 2010; Zhou *et al.*, 2013). Some H1 linker histones have been implicated in abiotic stress responses (Ascenzi and Gantt, 1997; Jerzmanowski *et al.*, 2000; Rutowicz *et al.*, 2015). In Arabidopsis, linker histones H1.1 and H1.2 are constitutively expressed while H1.3 is strongly induced by abiotic stress and not expressed under non-stress conditions (Ascenzi and Gantt, 1997; Rutowicz *et al.*, 2015). Chromatin changes also play a role in biotic stress responses (van den Burg and Takken, 2009; Palma *et al.*, 2010; Jaskiewicz *et al.*, 2011; Singh *et al.*, 2014). More recently it was discovered that the histone chaperone chromatin assembly factor 1 represses priming of plant defense response genes (Mozgová *et al.*, 2015). The plant defense response is coordinated by the accumulation of chemical signals and associated with extensive transcriptional reprogramming (Feys and Parker, 2000). One of those signals is salicylic acid (SA), which was first linked to the establishment of systemic acquired resistance and pathogenesis-related protein induction about 20 years ago (Delaney *et al.*, 1994).

In order to analyze whether other proteins related to chromatin compaction might be involved in priming or plant defense responses, transgenic Arabidopsis lines expressing H1-GFP fusion proteins were challenged with SA. To our surprise SA application led to a strong decrease in fluorescence signal. The signal loss was independent of protein abundance. A similar decrease was found for other GFP fusion proteins independent of their promoter or subcellular localization. The related fluorophores RFP and VENUS were also sensitive to SA. From our study we conclud that assays measuring relocation or turnover of GFP‐tagged proteins upon SA treatment should be evaluated very cautiously.

## Materials and methods

### Plant material and growth conditions

Wild type plants were *Arabidopsis thaliana* accession Columbia (Col-0). Transgenic lines were pH1.1:H1.1-EGFP, pH1.2:H1.2-EGFP, pH1.3:H1.3-EGFP (She *et al.*, 2013), p35S:H2A-GFP, pRPS5a:mDII-VENUS (Brunoud *et al.*, 2012), p35S:Tubulin‐A-smRSGFP (Ueda *et al.*, 1999) pMSI1:MSI1-mGFP4 (Derkacheva *et al.*, 2013) and p35S:U2B”- sGFPS65T (Boudonck *et al.*, 1999). The pUBQ10-mRFP line was generated using plasmid pUBC-RFP (Grefen *et al.*, 2010) for transformation of Arabidopsis plants by floral dip. Plants were grown on ½ Murashige and Skoog (MS) medium, including vitamins (Duchefa, Haarlem, The Netherlands), in squared plates (Sarstedt, Nümbrecht, Germany). Plates were sealed with micropore tape and stratified for 2 d at 4°C in the dark. The plates were then transferred to a growth chamber and grown vertically with a light-dark cycle of 16 h light at an intensity of 110 µmol m^-2^ s^-1^ at 22°C and 8 h dark at 20°C periods.

### Salicylic acid treatment and confocal microscopy

Ten-day-old seedlings were transferred into 6-well plates containing 5 ml of liquid ½ MS medium including vitamins and different concentrations of SA (Simga C St. Luis) ranging from 0–200 µM. SA was dissolved in DMSO. Mock treatments contained equivalent amounts of DMSO as SA treatments. After the indicated treatment times, rangig from 0.5–6 h, roots of seedlings were mounted in the incubation solution on microscope slides and green fluorescence signals were detected with a confocal microscope (LSM Zeiss 800). The settings of the microscope were adjusted for the *pH1.1:H1.1-GFP* control sample and all following images were taken with the same settings i.e. excitation wavelength 488 nm, emission wavelength 509 nm, detector gain 624 V, pinhole 32 µm, airy units 2.80. For *pRPS5:mDII-VENUS*, the settings were adjusted to: excitation wavelength 488 nm, emission wavelength 509 nm, detector gain 758 V, and pinhole 120 µm, airy units 2.80. For the *pUBQ10:RFP* the settings were adjusted to: excitation wavelength 590 nm, emission wavelength 612 nm, detector gain 824 V, and pinhole 74 µm, airy units 3.05. Images of *pMSI1:MSI1-GFP* were taken with a higher detector gain of 688 V. Settings for Fig. 2, 3 and 4 were adjusted for the control and subsequent images were taken with the same settings: for *pH1.1:H1.1-GFP*, excitation wavelength 488 nm, emission wavelength 509 nm, detector gain 604 V, pinhole 89 µm, airy units 2.80; for *pMSI1:MSI1-GFP*, detector gain 774 V; for *p35S:U2B”-GFP*, detector gain 710 V; for *p35S:H2A-GFP*, detector gain 588 V; for *pH1.2:H1.2-GFP*, detector gain 622 V; for *p35S:Tubulin‐A-GFP*, detector gain 707 V. For *pRPS5:mDII-VENUS,* settings were adjusted to: excitation wavelength 508 nm, emission wavelength 524 nm, detector gain 694 V, pinhole 92 µm and airy units 2.80. For the *pUBQ10:RFP* settings were adjusted to: excitation wavelength 590 nm, emission wavelength 612 nm, detector gain 745 V, pinhole 119 µm and airy units 3.05. Experiments were repeated at least two times. For *H1-GFP* constructs, experiments were repeated four times using three different batches of SA.

### Protein isolation and detection

Total protein was isolated from 200 mg of seedlings with Laemmli Buffer. Total proteins weighing 15 µg were separated by 12% SDS-PAGE and transferred to polyvinyl difluoride (PVDF) membranes (Roth, Karlsruhe, Germany) by wet blotting at 4°C in 25 mM Tris-HCl at pH 8.3, 24 mM glycine and 10% ethanol for 2 h at 250 mA. Enhanced chemiluminescence detection was performed using an ECL Western Blotting Substrate kit as recommended by the manufacturer (Thermo Scientific, Rockford, USA). Mouse anti-GFP antibodies [632381(JL-8), Clontech], at a dilution of 1:1000, were used for immunoblotting. Secondary antibodies were Alexa Fluor® 555 goat anti-mouse (Fig. 5A) SFX Kit (A31621, Invitrogen Life Technologies) and goat anti-mouse HRP (Fig. 5B) (170–6516, Biorad, Solna, Sweden). Western blots were performed independently three times and gave consistent results; one representative blot is shown.

### *Measurements of EGFP fluorescence* in vitro

Recombinant enhanced green fluorescent protein (EGFP) (Nordic BioSite, Sweden, Lot 614PEGFP) in phosphate-buffered saline (PBS) buffer at pH 7.4 (Nordic BioSite, Sweden,) was treated with 200 µM SA and 20% DMSO or only DMSO. Triplicates of a total volume of 100 µl, with 1 µg/ml EGFP, were pipetted into a black 96-well microplate (Nunc) for each time point. EGFP fluorescence was measured with a FLUOstar Omega fluorescence plate reader (BMG Labtech Germany, Offenburg). The excitation filter was set to 485 nm, emission filter to 520 nm and the gain was set to 2000. Fluorescence was measured and concentrations calculated based on a previously established standard curve.

## Results

### SA treatment decreases GFP signals in roots in a concentration-dependent manner

**Fig. 1.**
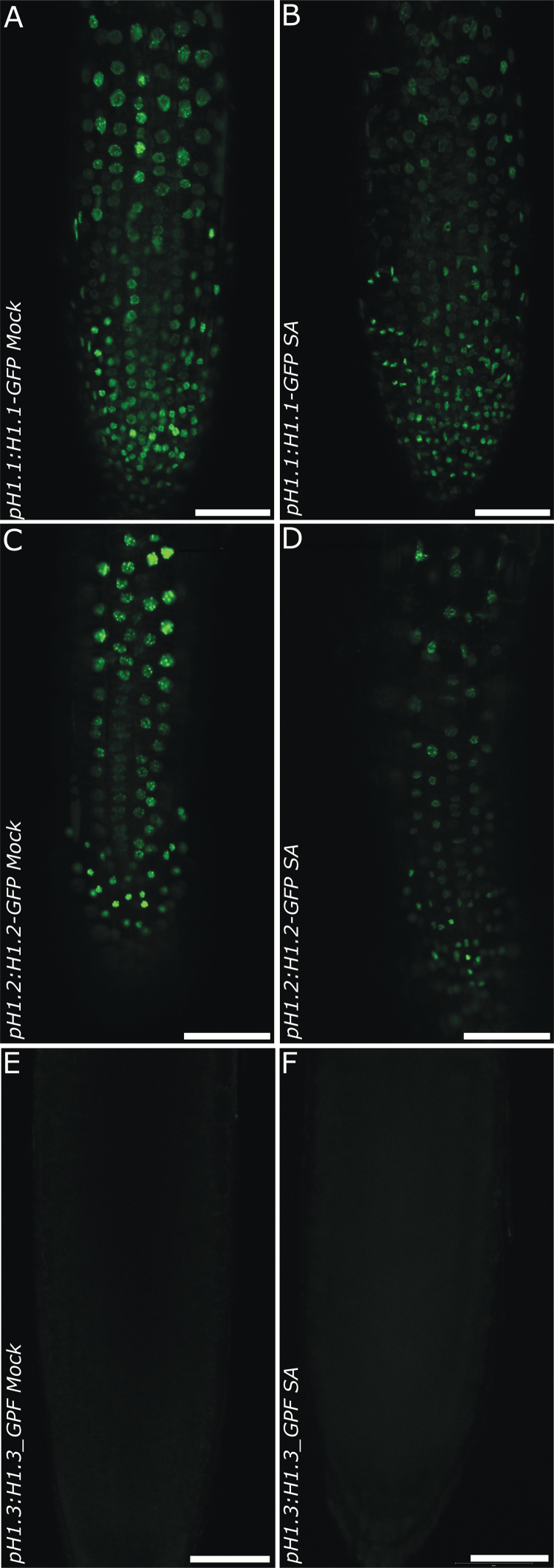
SA-dependent decrease of GFP signal in roots of plants expressing Histone H1 and H1.2 GFP fusion proteins. Seedlings of transgenic lines expressing *pH1.1:H1.1-GFP* (A-B), *pH1.2:H1.2-GFP* (C-D) or *pH1.3:H1.3-GFP* fusion constructs were either control treated (A, C and E labeled as Mock) or treated with 50 µM SA (B, D and F labeled as 50 µM SA). Images were taken after 1 h of incubation. Scale bar = 50 µm.

**Fig. 2.**
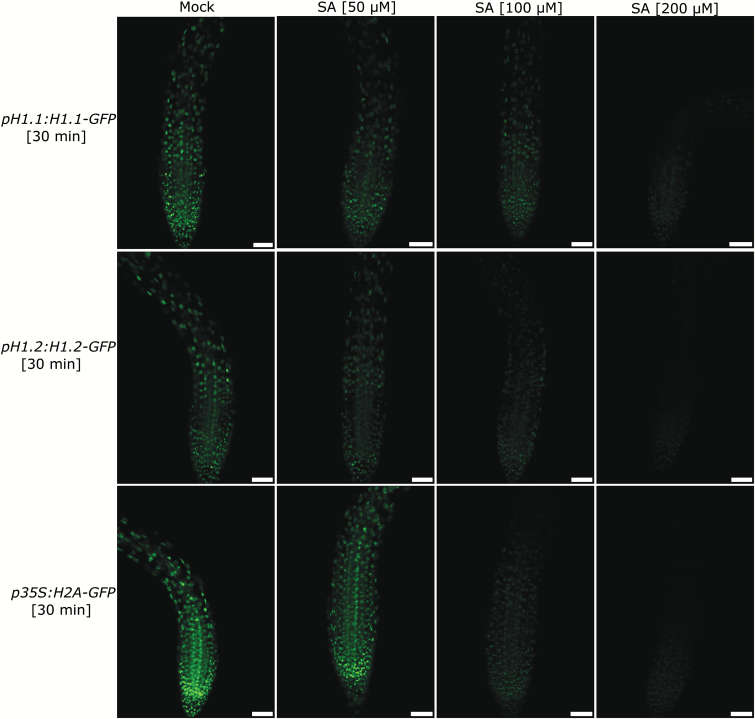
SA-dependent decrease of GFP signal is concentration dependent. Seedlings of transgenic lines expressing *pH1.1:H1.1-GFP*, *pH1.2:H1.2-GFP* or *p35S:H2A-GFP* fusion constructs were treated with 0 µM (first column), 50 µM (second column), 100 µM (third column) or 200 µM SA (last column). Images were taken after 30 min of incubation. Compare the partial loss of GFP signal after 50 µM to the complete loss after 200 µM SA. Scale bar, 50 µm.

To test the potential effects of biotic stress on linker histones we made use of transgenic lines carrying *EGFP* reporter constructs for H1.1, H1.2 and H1.3 (She *et al.*, 2013). We analyzed the effect of SA application on their expression levels and eventual localization changes. As expected, strong GFP signals were clearly visible in control nuclei for the H1.1 and H1.2 but not H1.3 constructs (Fig. 1A, C, E). In many nuclei, GFP-positive subnuclear structures could be observed that probably correspond to chromocenters (Fransz *et al.*, 2002). In contrast to controls, nuclei from plants incubated in liquid growth media containing SA showed decreased fluorescence signals (Fig. 1B, D). H1.3 is only expressed during drought and low light stress and we did not observe any expression induction under SA stress conditions within the time frame of our experiments (Fig. 1E, F). The fluorescence signal reduction observed for H1.1 and H1.2 reporters was concentration dependent, as 100 µM and 200 µM SA led to stronger reductions than 50 µM SA (Fig. 2). Although signal intensity was reduced, protein localization was unaffected in the H1.1 and H1.2 reporter samples treated with SA and remained restricted to the nucleus (Figs 1, 2). Furthermore, the localization was also independent of the SA concentration applied (Fig. 2). Linker histones stabilize the compact higher-order chromatin structure by binding inter-nucleosomal linker DNA (Kotlinski *et al.*, 2016). It seemed possible that a decrease in fluorescence signal was linked to disassociation of H1 from chromatin as a part of massive transcriptional reprogramming during a plant defense response.

In contrast to linker histones, nucleosomal histones cannot as easily be exchanged. We therefore asked whether it was only H1 and not core histones that form the nucleosomes that reacts to SA application. Histone H2A is one of the four histones that form the nucleosome octamer (Luger *et al.*, 1997). We treated transgenic Arabidopsis plants expressing a *p35S:H2A-GFP* fusion construct with SA as described before. In stark contrast to our expectations, the GFP signal also decreased upon SA treatment for the core histone H2A-GFP fusion (Fig. 2).

**Fig. 3.**
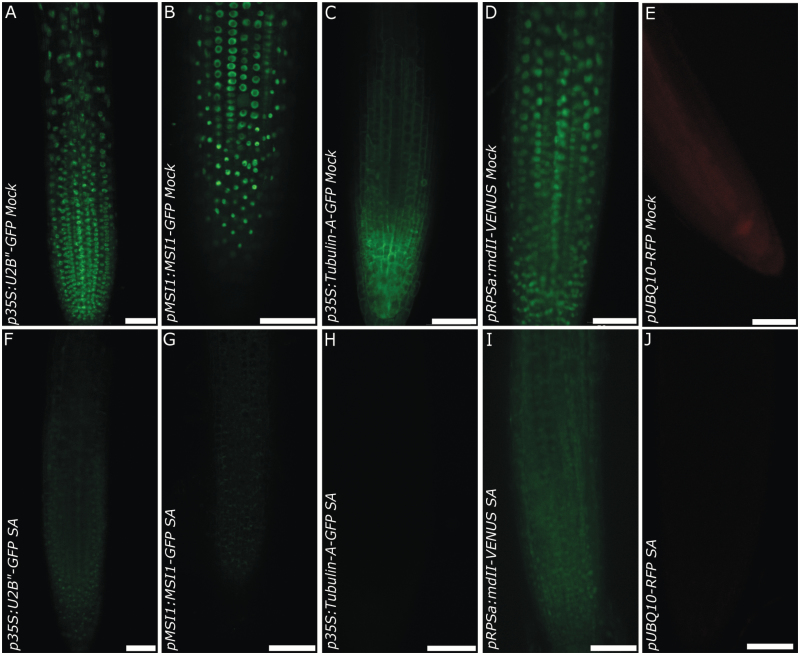
SA-dependent decrease of fluorescent protein signal is independent of the promoter or cellular localization of the fusion proteins. Transgenic lines expressing different GFP fusion constructs were mock-treated (upper images) or treated with 50 µM SA (lower images) for 1 h. Constructs used were: *p35S:U2B”-GFP* (A and F), *pMSI1:MSI1-GFP* (B and G), *p35S:Tubulin‐A-GFP* (C and H), *pRPS5a:mDII-VENUS* (D and I) and *pUBQ10:RFP* (E and J). Note that all reporter lines showed a similar sensitivity to SA. Scale bar, 50 µm.

**Fig. 4.**
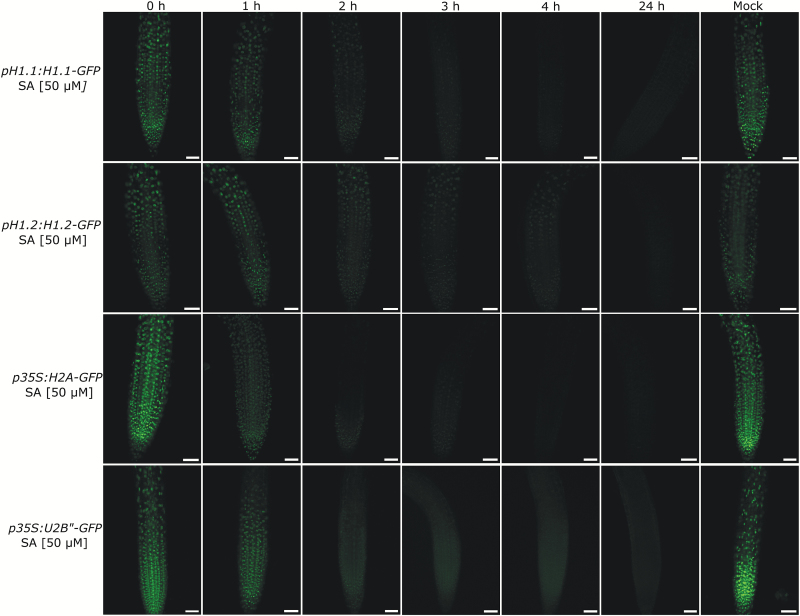
SA-dependent decrease of fluorescent protein signal with time. Seedlings of transgenic lines expressing *pH1.1:H1.1-GFP*, *pH1.2:H1.2-GFP*, *p35S:H2A-GFP* and *p35S:U2B”-GFP* fusion constructs were treated with 50 µM SA. Images were taken every hour after the start of incubation. Compare the partial loss of GFP signal after 1 h to the complete loss after 24 h. Scale bar, 50 µm.

It appeared highly unlikely to us that the bulk of nucleosomal core histones would be exchanged within one hour of SA treatment. We suspected that instead the fluorescence signal might be lost independently of the fusion partner. To test this hypothesis, we repeated the experiment with several other fusion proteins driven by diverse promoters and targeted to different subcellular compartments involving different fluorophores. We used microtubule-associated ectopic *p35S:Tubulin‐A-GFP*, auxin-sensitive *pRPS5a:mDII-VENUS,* ectopic cytoplasmic and nuclear *pUBQ10:RFP,* the splicosomal associated *p35S:U2B”-GFP* and the histone binding protein *pMSI1:MSI1-GFP*. Treatment with SA reduced fluorescence signal strength in all lines independent of fusion protein, promoter, intracellular localization or fluorophore (Fig. 3). Note that the microtubule association of tubulin-A-GFP is better resolved in a close-up of a lateral root (see Supplementary Fig. S1 at *JXB* online). In the case of *pRPS5a:mDII-VENUS*, which showed mostly nuclear but also some cytoplasmic localization in control samples, SA treatment appeared to reduce the nuclear signal faster than the cytoplasmic signals or to induce partial intracellular relocalization in some cells (Fig. 3D, I). No other reporter constructs showed any effects of SA on their protein localizations. At a concentration of 50 µM SA, a reduction in fluorescence signal was only seen in some lines when the treatment lasted no more than an hour (Figs 1–3). However an extended incubation of 3 h led to a total loss of signal (Fig. 4). This indicates that SA concentration mainly affects the rate of fluorescence decrease.

Taken together, the treatment of Arabidopsis plants with 50 µM SA can lead to rapid loss of fluorescence for a range of fluorescent reporters *in vivo*.

### GFP fusion protein abundance is unaffected by SA treatment

**Fig. 5.**
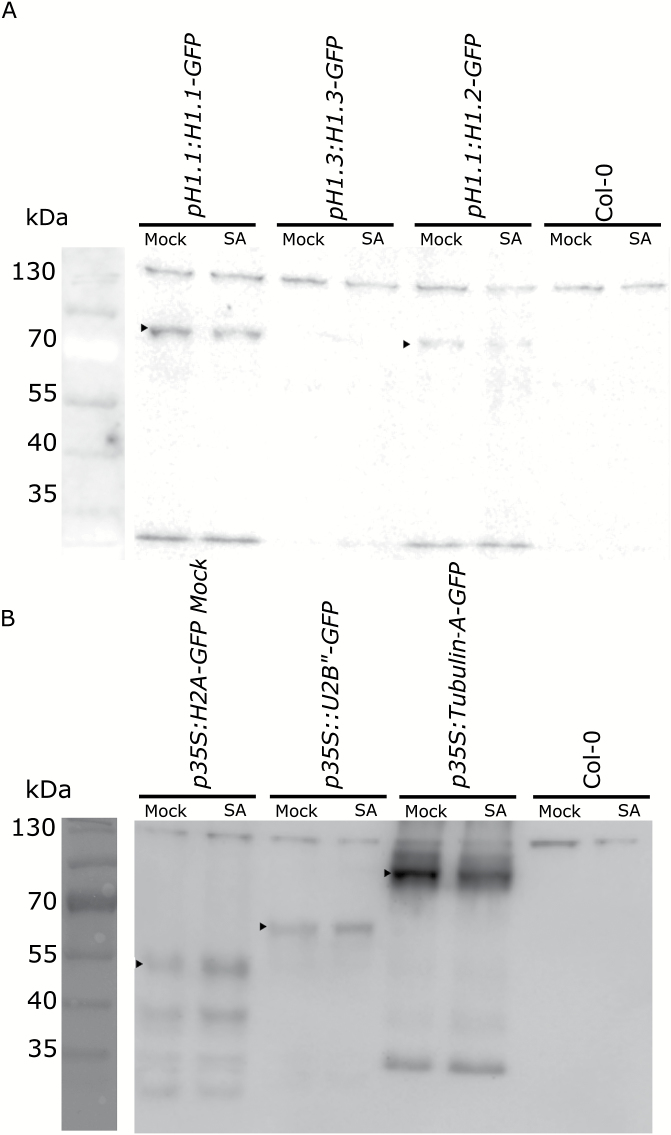
GFP fusion protein abundance is unaffected by SA treatment. Immunoblot analysis from mock-treated and 200 µM SA-treated plants using anti-GFP antibodies. Samples were taken after 1 h. (A) 10% SDS-PAGE of 18 µg total protein from *pH1:H1-GFP* (~64 kDa), *pH1.3:H1.3-GFP* (~50 kDa), *pH1.2:H1.2-GFP* (~64 kDa) and Col-0 control plants. (B) 12% SDS-PAGE of 10 µg protein from *p35S:H2A-GFP* (~32 kDa)*, p35S:U2B”-GFP* (~52 kDa), *p35S:Tubulin‐A-GFP* (~75 kDa) and Col-0 control.

Proteins fused to GFP have been widely used as transcriptional and translational markers to assess the timing of transcription as well as protein expression (Chalfie *et al.*, 1994). GFP is relatively stable in the cell under a wide pH range of 2–12, (Vrzheshch *et al.*, 2000), although fluorescence is impaired in acidic compartments such as the vacuole (Tamura *et al.*, 2003). We therefore tested whether the loss of fluorescence signal is caused by protein turnover. We performed protein immunoblot analysis with proteins isolated from plants expressing various fusion proteins that were mock- or SA-treated. In contrast to the complete loss of GFP signal in 200 µM SA, no strong difference in protein abundance could be detected between treated and mock samples (Fig. 5A). All transgenic lines showed similar results independent of their promoter or subcellular localization (Fig. 5B).

Together, these results established that SA does not initiate rapid degradation of fluorescent reporters but can interfere with reporter fluorescence.

### In vitro *EGFP fluorescence is stable in the presence of SA*

**Fig. 6.**
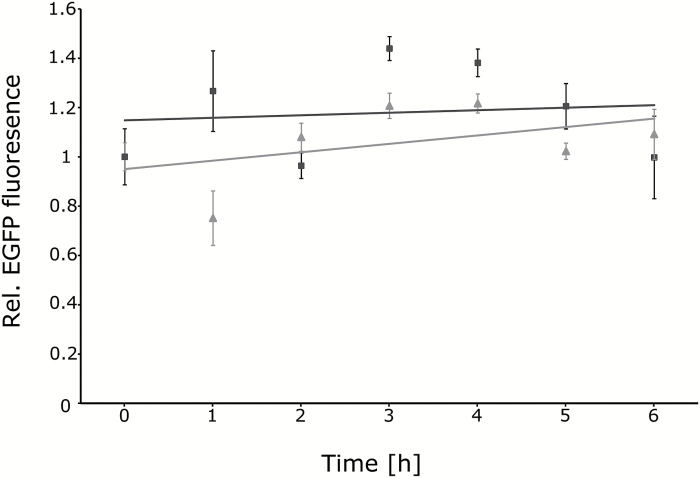
*In vitro* fluorescence of EGFP remains stable in the presence of SA. Fluorescence of recombinant EGFP protein either mock-treated (grey diamonds) or treated with 200 µM SA (black squares) was measured over a time course of 6 h. Each data point represents the average of three replicates. Error bars indicate SE.

To test whether SA affects GFP fluorescence directly, we measured the effect of SA on EGFP fluorescence *in vitro*. Recombinant EGFP was incubated with 200 µM SA, which rapidly reduced reporter fluorescence *in vivo*. The fluorescence was measured for up to 6 h of incubation. Neither in mock- nor in SA-treated samples was a reduction in EGFP fluorescence detected (Fig. 6). This result strongly suggests that SA does not directly affect GFP fluorescence but rather requires cellular components to cause fluorescence reductions.

## Discussion

The use of fluorescent proteins in molecular biology as protein tags has helped decipher diverse molecular pathways. Since the discovery of GFP and its ability to be fused to almost any protein of interest, GFP has been used in countless studies. Nevertheless, even a technique as commonly used in molecular biology as GFP-tagging has its limitations. In an attempt to understand whether linker histones are involved not only in abiotic but also biotic stress responses, we identified a surprising reduction in fluorescence signal of GFP-tagged fusion proteins following SA treatment. SA is a signaling molecule in plant immune responses. It is produced after pathogen recognition to activate defense genes that may lead to resistance (Vlot *et al.*, 2009; Fu and Dong, 2013).

Approximately 1 h after the application of SA, the previously clearly visible GFP signal in the nucleus of transgenic lines carrying *pH1.1:H1.1-GFP* or *pH1.2:H1.2-GFP* fusion constructs decreased strongly. A similar SA-induced reduction of fluorescence was observed for H2A-GFP and other reporters. We observed SA-induced reduction of fluorescence for nuclear (*pH1.1:H1.1-GFP*, *pH1.2:H1.2-GFP*, *p35S:H2A-GFP*, *pMSI1:MSI1-GFP*), microtubule-associated (*p35S:Tubulin‐A-GFP*) and cytoplasmic (*pUBQ10:RFP*) proteins. Six different promoters (*pH1.1*, *pH1.2*, *p35S*, *pRPS5a, pUBQ10, pMSI1*) were used and showed similar sensitivity to SA. Finally, the reduced fluorescence was observed for three different fluorescent reporters: GFP, RFP and VENUS. Thus, the reduction of fluorescence signals after SA application is independent of the promoter driving the reporter and the location and nature of the fusion proteins. This effect was also seen for VENUS and RFP fluorophores, illustrating that it is not specific for GFP. mDII:VENUS is a sensor for auxin response and distribution (Brunoud *et al.*, 2012). Loss of mDII:VENUS signal can be induced by auxins. SA has been reported to inhibit auxin signaling but not to affect free auxin concentrations in the plant (Wang *et al.*, 2007). We therefore consider it to be very unlikely that SA affected mDII:VENUS via auxin-induced protein turnover.

*In vitro* experiments with recombinant EGFP showed that SA caused GFP fluorescence reduction only *in vivo* but not *in vitro* under our experimental settings. SA is produced by many plants including Arabidopsis and the applied concentration of 50 µM is within the range of values commonly used in SA experiments. Even at up to 5 mM SA Arabidopsis seeds still germinate (Kang and Klessig, 2005; Rivas-San Vicente and Plasencia, 2011). Thus, we consider it unlikely that SA toxicity caused cell death and therefore fluorescence reduction. This and the stability of the reporter proteins indicate that the effect is on the fluorophore or fluorescence itself. We note that GFP has been successfully used in SA studies before (Boursiac *et al.*, 2008; Love *et al.*, 2012; Du *et al.*, 2013), indicating that the reliability of GFP may depend on experimental settings, in particular SA concentration and duration of exposure. For instance, we observed only mild fluorescence reduction for some reporter lines after 30 min at 50 µM. Medium composition, SA application strategies and metabolic conditions could also affect final intracellular SA concentrations.

Considering the widespread application of GFP-tagged fusion proteins in diverse fields including stress biology, great care must be taken when utilizing fluorescent reporters to avoid misleading results and prevent erroneous conclusions.

## Supplementary Material

Supplementary Data

## Abbreviations:

EGFP: enhanced green fluorescent protein
GFP: green fluorescent protein
MS: Murashige and Skoog
PBS: phosphate-buffered saline
PVDF: polyvinyl difluoride
RFP: red fluorescent protein
SA: salicylic acid
