# Supplementary material for: Salicylic acid interferes with GFP fluorescence in vivo
Source: J Exp Bot. 2017 Mar 29;68(7):1689–96. doi: 10.1093/jxb/erx031 (PMC5441896; doi:10.1093/jxb/erx031)
Supplement: Supplementary Data [file erx031_Supplementary_Data.zip › supplementary_figure_S1.pdf]

Salicylic acid interferes with GFP fluorescence *in vivo*

Jennifer de Jonge, Daniel Hofius, Lars Hennig

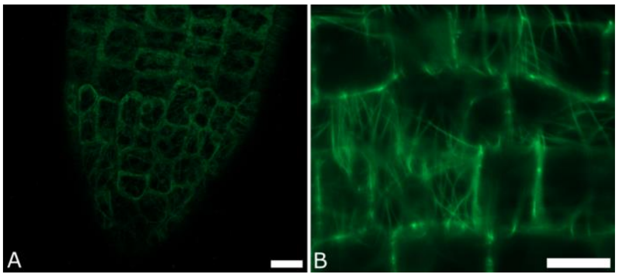

Supplementary Fig. S1. Microtubule organization in lateral roots of control plants

(A) Lateral root tip of p35S:Tubulin-A-GFP plants showing microtubule organization across the cells. (B) Close up of lateral root epidermal cells showing microtubule organization. Scale bar, 10 μM.
